# 4319 Team Science in Parkinson’s Research: Connecting Clinicians and Computational Teams

**DOI:** 10.1017/cts.2020.359

**Published:** 2020-07-29

**Authors:** Luba Smolensky

**Affiliations:** 1 The Michael J Fox Foundation

## Abstract

OBJECTIVES/GOALS: This team science pilot program aims to elevate the quality of Parkinson’s disease modeling initiatives by strengthening connections between clinical researchers and computational teams. As many data science projects in Parkinson’s research would benefit from deeper clinical expertise, many clinical engagements would be improved by upfront integration of computational requirements. These team science programs, developed from design thinking methodologies, provide structured, sustainable, and scalable means for multi-disciplinary teams to come together and co-create translational science in PD. METHODS/STUDY POPULATION: Design Thinking (DT) could help yield an effective learning experience. DT is grounded in ethnographic research strategies and prototyping, relying heavily on grantee interviews and feedback. This approach is commonly used to navigate and design amidst complexity; its applications range from product to healthcare to instructional design. The following is an overview of the process as applied to this project: *Discover*: Once the core team (MJFF and project designers) has refined the key question they would like to answer, the team will begin gathering both primary and secondary data. This phase may include focus groups, one-on-one interviews, expert interviews, and immersive data-gathering. The purpose of this phase is to capture complexity and lay the groundwork to understand grantees’ perspectives and lexicon around their work. The deliverables of this phase are primarily unstructured research findings, such as transcribed interviews and secondary sources. *Define*: When sufficient data has been gathered, the core team will move into an initial round of synthesis and sense-making (making connections and assumptions to explain emerging themes in the data). This phase may include one to two in-person engagements with the core team. The purpose of this phase is to define the guiding principles for subsequent prototypes. It will also help reveal potential opportunity areas, both latent or apparent. The deliverables of this phase are agreed upon key themes, insights, and an informed “How Might We” question that will anchor the ideation process. *Develop*: Armed with informed themes, the core team will begin to brainstorm potential solutions. Following a set of brainstorming techniques, they will initially aim for quantity versus quality in order to allow potentially innovative and/or risky solutions to surface. Eventually, these ideas will be distilled into three robust and unique prototypes. Like the prior phase, ideation may also require one to two in-person engagements. The deliverables here are three unique prototypes; the reason for three is the ensure that the team does not anchor themselves in just one solution, but rather remains in an exploratory mindset as they solicit feedback on these prototypes from the grantees. *Deliver*: In this final phase, the core team revisits the grantees and presents the three prototypes. This phase may include conducting three small-scale pilots or simply just explaining the prototypes. Either way, it is important to solicit another round of feedback to ensure the solutions are indeed addressing the needs and context of grantees. Once completed, the core team will iterate a final pilot design and identify any remaining questions and assumptions they would like the pilot to inform. RESULTS/ANTICIPATED RESULTS: The team science pilot identifies five main opportunities to tighten collaboration, communication, and expectations across clinical and computational teams. Firstly, in-person events, held regularly in a central location, can act as an incubating space for these teams to partner, ideate, and pitch for grant funding. Secondly, co-developed guidelines for research questions would ensure consistent availability of clinically-relevant, computationally-feasible research topics. Thirdly, increasing the presence of Parkinson’s cohort data resources at computational conferences could introduce more diverse data and genetics interest in Parkinson’s research. Fourthly, a standard suite of research-facing, educational content (focused on both disease background and data basics) would ensure a strong baseline and launch-pad for PD modeling projects. Lastly, a fellowship program focused on early-stage researchers could establish a unique foundation to ground both clinical and computations fellows to collaboratively work on PD research as well as iterate on the aforementioned solutions. DISCUSSION/SIGNIFICANCE OF IMPACT: This team science program has the potential to upend collaborative silos in Parkinson’s research, accelerating disease modeling projects which otherwise stagnate or over-emphasize clinical v. computational aspects. By more effectively connecting team members with diverse backgrounds across clinical and computational roles, PD disease patterns can be discovered and validated ultimately resulting in improved patient care and therapeutic development. CONFLICT OF INTEREST DESCRIPTION: Several authors are staff members at The Michael J. Fox Foundation for Parkinson’s Research, the sponsor of this Team Science grant. All author and non-author contributors are grant recipients from The Michael J. Fox Foundation.

